# Increasing the Capacity of Primary Care Through Enabling Technology

**DOI:** 10.1007/s11606-016-3952-3

**Published:** 2017-02-27

**Authors:** Heather M. Young, Thomas S. Nesbitt

**Affiliations:** 1 Betty Irene Moore School of Nursing, UC Davis Health System, Sacramento, CA 95817 USA; 2 UC Davis Health System, Davis, CA USA; 3 Family and Community Medicine, UC Davis, Davis, CA USA; 4 Center for Information Technology Research in the Interest of Society, University of California, Davis, CA USA

**Keywords:** technology, primary care, mHealth, telehealth, sensors, patient portal

## Abstract

Primary care is the foundation of effective and high-quality health care. The role of primary care clinicians has expanded to encompass coordination of care across multiple providers and management of more patients with complex conditions. Enabling technology has the potential to expand the capacity for primary care clinicians to provide integrated, accessible care that channels expertise to the patient and brings specialty consultations into the primary care clinic. Furthermore, technology offers opportunities to engage patients in advancing their health through improved communication and enhanced self-management of chronic conditions. This paper describes enabling technologies in four domains (the body, the home, the community, and the primary care clinic) that can support the critical role primary care clinicians play in the health care system. It also identifies challenges to incorporating these technologies into primary care clinics, care processes, and workflow.

Primary care plays an essential role as first point of contact with the health care system and as a vehicle for delivering continuous, comprehensive, and coordinated care. It may be particularly relevant for addressing problems faced by individuals with chronic illnesses and multiple comorbidities.^1^^,^ 2 However, as currently structured, primary care faces a number of challenges, including difficulty in securing the clinical, information, and financial resources needed to improve the patient experience, optimize population health, and ensure a viable future for the field.^3^ Primary care is well positioned to use technology to disrupt current models and to enable care in the right place at the right time. Technology, appropriately used, unleashes new possibilities for addressing pressing issues of efficient and effective care delivery and promoting healthy aging in the community.^4^^,^ 5 Adoption of technology in primary care, however, is stymied by a number of factors, including expense, culture change, disruption in care processes and workflow, training requirements, and competing priorities for practice improvement.

The first part of this paper identifies a variety of enabling technologies that could increase capacity in primary care to enact the Institute of Medicine (IOM) vision of “providing integrated, accessible health care services by clinicians who are accountable for addressing a large majority of personal health care needs, developing a sustained partnership with patients, and practicing in the context of family and community.”^6^ The second part discusses the challenges to achieving this vision and logistical issues for primary care.

## THE TECHNOLOGY TOOL BOX—EXTENDING THE REACH

**Figure 1:**
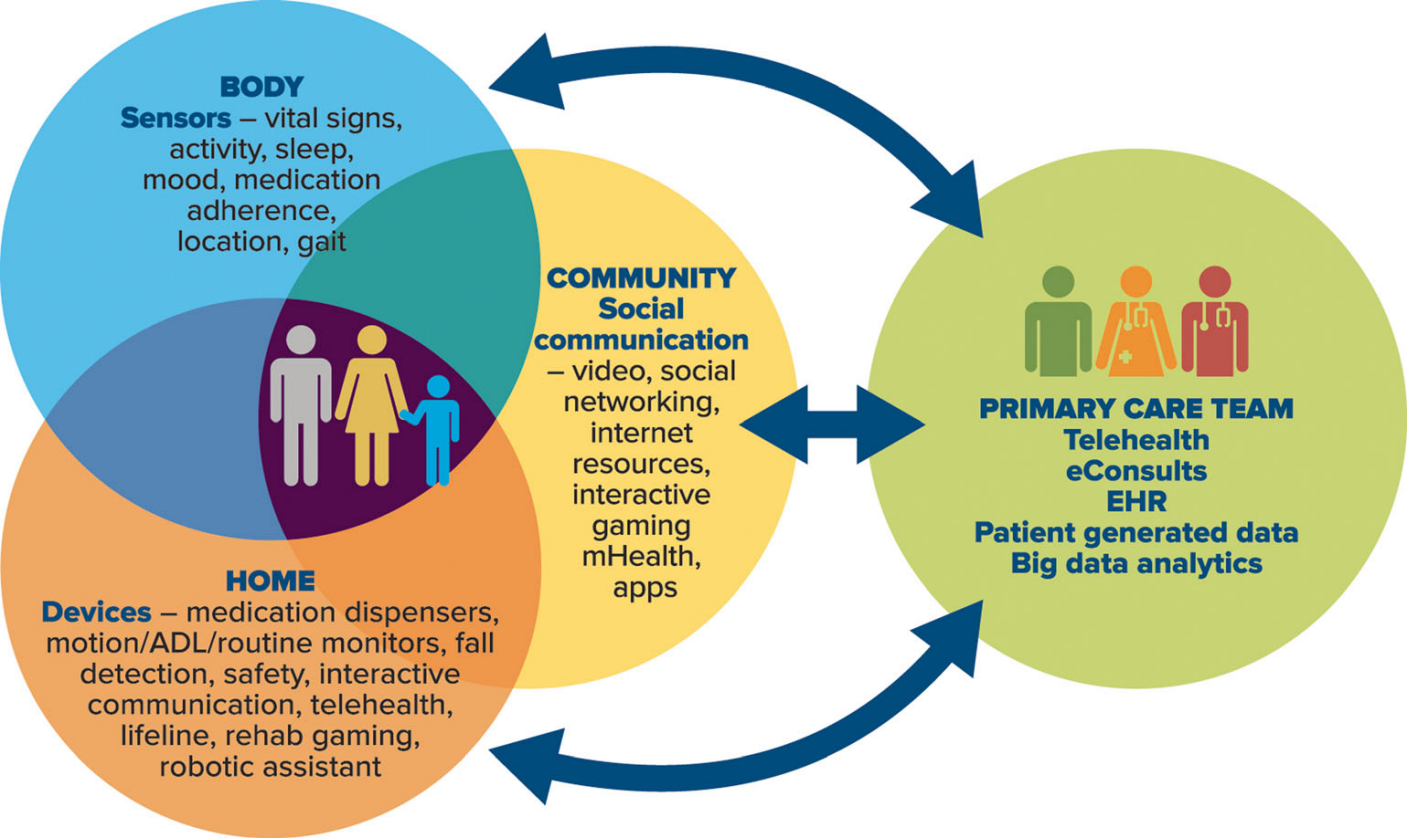
Technology-enabled primary care.

A variety of technological advances hold promise for increasing capacity and quality in primary care. Figure 1 highlights four domains of technology most pertinent to primary care, including technology tethered to the body, the home, the community, and the primary care clinic. Technology offers the potential to collect relevant patient-generated data, aggregate clinical data, and facilitate communication among all members of the care team regarding these data, bolstered by pertinent expert knowledge and evidence. Examples of some of the more promising technologies are discussed below.

### The Body

Body sensors and monitors include an array of data collection devices that could be useful in managing health conditions outside traditional clinical settings.^7^ Industry estimates predict that the annual market volume for smart wearable health care will grow from $2 billion in 2014 to $41 billion in 2020,^8^ with over 80% of consumers saying that an important benefit of wearable tech is its potential to make health care more convenient.^9^ In addition, 70% of adults track some health parameter, such as blood pressure, for themselves or a family member.^10^

Target populations for body sensors and monitors are patients with chronic conditions requiring monitoring, undergoing changes in medication, at risk for preventable hospitalization, or needing motivation for health behavior change. Target parameters include vital signs, sleep, blood glucose, oxygen saturation, activity patterns, location, gait, and signals of ingestion of prescribed medication. These technologies generally feature sensor devices that connect to wireless communications and local storage that has an interface with a centralized data repository and/or the electronic health record, where data are graphically displayed as actionable information for the clinician, and where diagnostic analytics are incorporated for interpretation of patterns and development of either treatment recommendations or intervention alerts.

Patient-generated data can provide relevant clinical information in the context of the home or work environment and in relation to associated routines and activities, giving the clinician real-time information about physiological and/or behavioral patterns and responses to treatment. While there are concerns about the accuracy and reliability of some off-the-shelf apps and devices,^11^ there is growing evidence of the benefits of remote monitoring in several chronic conditions.^12^^–^ 17 For example, patient engagement through a combination of paired glucose testing and nurse coaching resulted in substantial improvements in glycemic control among individuals with diabetes.^18^^,^ 19 (See Text Box 1 for an illustrative example.) Mobile health applications and body sensors have a number of benefits, including increased capacity on the part of individuals for self-management as they receive feedback regarding their responses, and increased provider capacity to be responsive at the time of need, enabling just-in-time intervention rather than during a later clinic visit.^20^

**Text Box 1:** Technology Improving Health in Diabetes

LG is a 35-year-old Latina woman with type II diabetes, BMI of 31.5 and HbA1C averaging 9.5. Despite implementing a meal plan recommended by the dietitian and walking 30 min every day, her lab values remain in an unacceptable range. At times she forgets to take her oral hypoglycemic agent. She is highly motivated to change her behavior and is open to new ideas. She has a cell phone and has been testing her blood glucose in the morning several times a month.
The nurse in the primary care clinic meets with her to understand her goals and her barriers to achieving her goals. They agree to try new strategies for a month-long trial. Given LG’s goals to increase her physical activity and improve her dietary intake of fruits and vegetables, they select the following tools, all connecting to her iPhone and using iHealth to collect and summarize the data:
• app to track dietary intake
• app to track medications
• activity tracker to record steps, sleep and activity patterns
• blood glucose monitor for paired glucose testing before and after planned exercise and selected meals
Each week they meet to review the data together and to discuss trends and patterns. At the end of a month, they identify the impact of specific dietary choices and patterns and type of physical activity on her blood glucose patterns and prioritize her strategies going forward.

Ingestible sensors embedded in medications use a digestible chip that signals that a specific capsule or tablet has entered the stomach. These devices communicate through a wearable patch and a mobile phone interface, and have shown promising early results. They may be particularly useful for complex and long-term medication regimens (such as with human immunodeficiency virus, tuberculosis, diabetes, or severe psychiatric illness), where adherence is critical.^21^^–^ 24

### The Home

The vast majority of older adults want to remain at home as long as possible, and many are turning to home-based enabling technology such as sensors and assistive devices as tools to achieve this goal.^5^ Smart homes are equipped to detect motion for the purposes of security, fall prevention, or recognizing deviations from routines. They may include motion detectors or cameras; bed sensors that can detect occupancy, motion, and body weight; and sensors on doors, drawers, and medication dispensing sets to detect use. Data collected can provide valuable information for understanding symptom patterns, activity, and responses to treatment, particularly in comparison to data elicited through recall unaided by diary records, which can be inherently inaccurate and acontextual.

Medication management is an integral part of chronic disease management, requiring the active involvement of both clinicians (whose role is to ensure appropriate prescribing and monitoring of effectiveness) and the individual (whose role is adherence and awareness of reportable symptoms). Technology solutions abound to aid in assessing indications, prescribing, dispensing, organizing, reminding, and monitoring effectiveness.^25^ Deployment of these tools can reduce the risk of adverse events and promote appropriate medication use.

Acute rehabilitation is also moving to the home.^26^ Off-the-shelf gaming technologies capture motion accurately and have been used for physical assessment and rehabilitation.^27^^,^ 28 Technology is also supporting and advancing function. Home-based sensors can adjust lighting and temperature, assistive technologies and robotics can improve mobility and performance of activities of daily living, and wireless or video-based programs can deliver structured rehabilitation protocols.^29^

### The Community

Individuals are turning to the internet for health information as well as engagement with others who have similar health conditions. Such digital engagement heralds the era of the ePatient,^30^ where attention to improving health occurs at all times rather than only during clinic visits. Thirty-five percent of US adults have gone online to research a health condition, and 41% confirmed their own diagnosis upon consulting with a clinician.^10^ Smartphone use is on the rise, and half of smartphone users have sought health information via their phones, with 20% downloading an app to monitor some aspect of their health. Web-based communities, such as PatientsLikeMe (https://www.patientslikeme.com/) and those provided by non-profit groups such as the Alzheimer’s Association, offer both information and connection with others sharing common conditions. Internet support groups, sponsored and moderated by individual clinicians or groups of clinicians working within consortia such as accountable care organizations, expand capacity and provide a resource for increasing patient knowledge, confidence, and coping with health conditions.^31^

### The Clinic

#### Telehealth

Telehealth enhances capacity by moving the expertise to primary care clinics rather than moving patients to the expertise. Interactive and asynchronous specialty consultation has been studied extensively over the past 20 years in multiple specialties and has demonstrated high patient satisfaction and quality.^32^^–^ 34 Telehealth can be deployed in several ways: between providers (e.g., primary care, specialists, diabetes educators) and patients, as group rounds, and between primary care team members and the patient in the home. All of these approaches bring expertise to the patient at the point of care, overcoming spatial and temporal barriers. They can be structured in various ways, from e-consults within the electronic health record, to asynchronous store-and-forward approaches (such as with retinal examinations or digital dermatology images), to live interactive conversations between health care professionals and/or with the patient. These consultations have the benefit of including the primary care provider (PCP) directly in the conversation with both the specialist and the patient, enhancing coordination of care and increasing PCP knowledge and capacity to manage the current patient as well as future patients presenting with similar conditions.

This model of care can reduce redundancy by keeping the PCP at the center of the encounter. Shared records streamline the process of consultation by allowing specialists to build upon a concise, relevant patient history and assessment presented by the PCP, and focus the specialist on adding value by getting to the next evidence-based decision. It also allows the PCP to be more integrated in follow-up, treatment implementation, and patient education. The use of electronic referrals (eReferrals) has enhanced communication between PCPs and specialists in comparison to paper-based communications, with significant improvement in specialists’ understanding of the consultation request, higher rates of appropriate referrals, greater identification of avoidable visits, and fewer required follow-up visits.^35^ Telehealth can also increase primary care capacity by broadening the clinical team to include social workers, nutritionists/dieticians, diabetes educators, pharmacists, and nurses on an on-demand basis. A current drawback to telehealth is that it relies on sight and sound, which can limit its usefulness when complex examinations involving palpation or other hands-on examination techniques are required. With the increase in diagnostic tools such as ultrasound, some of these limitations can be overcome. A systematic review of the evidence over the past decade supports the feasibility and acceptability of telehealth in primary care, with outcomes at least as good as usual care, and early indications of cost savings.^36^

Telehealth significantly reduces geographic disparities in specialty access, while contributing to education and professional development for practicing clinicians.^37^ Project ECHO demonstrated the power of distance education combined with group consultation, virtual rounds, and telehealth consultations in optimizing primary care for persons with hepatitis C, enabling patients to remain within their health care home, enriched by specialist involvement.^38^ This effort changed the approach to treating a condition that had historically required patients to go to major specialty centers for care, and has been successfully expanded to multiple other specialty areas.^39^ This approach has also brought psychiatric care to place-bound patients, as in rural communities, prisons, and nursing homes.^40^

#### Patient Portals and the Electronic Health Record (EHR)

The patient portal component of the electronic health record can enhance communication by making clinical information, assessments, and results available to patients, can provide secure messaging for simple questions to providers, facilitate medication refills, and coordinate appointments and billing activities.^36^ Patient education can also be tailored and delivered through these portals. With the integration of patient-generated data, the electronic health record becomes an even more powerful tool, as clinical data, treatment decisions, and patient behaviors can be considered simultaneously. Bidirectional communication and data sharing with the ability to visualize data summaries can improve partnership with individuals and families and enhance motivation for health behavior change.

## TECHNOLOGY CHALLENGES

While possibilities abound for technology-enabled care, the feasibility, usability, and evidence of its effectiveness lags. Body sensors, monitors, apps, and web-based programs, developed as consumer products, are proliferating outside the medical device development process, with its rigid focus on elements such as accuracy, privacy, and security. The accuracy and reliability of data is a major concern, as evidenced in a recent evaluation of a widely available unregulated app to measure blood pressure: the device demonstrated unacceptable sensitivity (0.22) and specificity (0.92) for detecting hypertension.^11^ As the market matures, developers must address barriers such as connectivity, reliability of sensors and apps, and reimbursement in order to enhance adoption in care delivery.^20^ The field would benefit from the development of validated app and device formularies from which the PCP could prescribe technology.

Even when sensors and devices are accurate and reliable, there are challenges to incorporating the data generated by these wearable devices into primary care practice. Data must be converted into actionable and relevant information that is easily accessible to the PCP within normal workflow, while minimizing false alarms. Although a number of efforts are under way in this area, standard interface platforms do not yet exist for all wearable devices and sensors. Clinicians are understandably concerned about their responsibility to act on abnormal data generated by wearables in real time and imported into the electronic health record, possibly increasing liability, and certainly affecting work flow.

Patient factors also play a role in the adoption of technology, including concerns about privacy, security, data sharing, the meaning data hold for the patient, and the context in which the data are generated and used.^41^ For a small proportion of patients, ready access to health data and a continuous focus on disease management can increase anxiety and offset any benefits that might accrue from intensive monitoring.^42^

Beyond issues of usability and adoption, health information technology remains out of reach for many Americans. Approximately half of older adults use the internet, and over three-quarters use cell phones, yet only 5–16% of Medicare beneficiaries use digital health technologies.^43^ This is compounded by a persistent digital divide, with more educated and affluent adults having greater access to technology.^44^ However, as the efficacy of remote monitoring and telehealth communication is established, it is possible that the cost barriers could be removed if payers incentivize the use of technology to enhance outcomes.

Technology is merely an enabling tool. Effective chronic disease management entails engaging patients in personal goal-setting and motivating behavior change. Large, well-controlled studies testing the contributions of technology to managing chronic disease are scarce and yield mixed results. A randomized controlled trial addressing diabetes, hypertension, and arrhythmia using smartphone-enabled biosensors failed to show a reduction in health care utilization or cost, but did demonstrate improved patient engagement in health self-management.^45^ Similarly, a large trial addressing heart failure yielded no cost benefits, but improved quality of life.^46^ Greater success has been realized with a comprehensive approach based on the Chronic Care Model, using care managers located within multi-payer primary care clinics, and deploying technology to provide individualized decision support and convenient communication among the patient and the team. This program has demonstrated both improved patient outcomes and improved provider productivity.^47^

## FUTURE DIRECTIONS: THE PATH FORWARD FOR TECHNOLOGY-ENABLED PRIMARY CARE

While technology holds great promise for improving health and health care, the adoption of technology in primary care is stymied by a number of factors, including perceived value, expense, incorporation into care processes and workflow, training requirements, disruption of established practices, and competing priorities for practice improvement.^48^ At the core of successful implementation is a strong strategy for intended use and outcomes, and a thoughtful plan for the required systemic changes in the organization.^49^ Primary care practices, therefore, should examine the potential benefits of enabling technology in light of the population served, the profile of health conditions amenable to technological support, and the readiness on the part of clinicians to invest time and resources in deployment. For example, body sensors can augment care in cases where traditional approaches are not producing desired outcomes, when the PCP needs additional data about adherence, and where patients might benefit from reinforcement for behavior change. Technology simply automates existing processes, and implementation often reveals issues in workflow.^50^ Therefore, prospective analysis of intended use and processes increases the likelihood of integration and actual improvements in efficiency.

In a systematic review of electronic health record implementation, several factors emerged as most significant in affecting outcomes, including design and functional features of the technology, project management, previous experience, and the “fit” of the technology with the social environment. Common concerns were privacy, security, safety, quality of care, staff anxiety, impact on provider/patient relationships, time, costs, efficiency, and liability.^50^ Large integrated health systems with chronic disease management teams and call centers will have fewer barriers to adoption of enabling technology; however, clinicians in small practices will have greater challenges. Smaller clinics might partner with home health agencies to monitor and triage incoming data for priority action by the PCP.

A technology-enabled health care delivery model has the potential to increase the role and relevance of primary care as the integrative hub for health care, supporting the efforts of patients, promoting collaboration with specialists, facilitating connections with nursing homes and home health, and advancing population health within a framework that improves the patient experience, quality, and value. Achieving this will require better preparation for patients and family caregivers, more training and professional development for clinicians, more systematic deployment of the technology tools themselves, and changes in policy and reimbursement to recognize the tools’ value. Efforts are under way to address improvements to technology and its deployment.^51^ Simultaneously, primary care, through professional development and practice redesign, must reimagine its role, contribution, and potential within the context of a technology-enabled delivery system.^52^
